# Roles and Functions of ROS and RNS in Cellular Physiology and Pathology

**DOI:** 10.3390/cells9030767

**Published:** 2020-03-21

**Authors:** Neven Zarkovic

**Affiliations:** Rudjer Boskovic Institute, Laboratory for Oxidative Stress (LabOS), Bijenička 54, HR-1000 Zagreb, Croatia; zarkovic@irb.hr

**Keywords:** free radicals, redox balance, cell signaling, growth, toxicity, antioxidants, oxidative homeostasis, oxidative metabolism of the cells, pathophysiology of oxidative stress

## Abstract

Our common knowledge on oxidative stress has evolved substantially over the years, being focused mostly on the fundamental chemical reactions and the most relevant chemical species involved in human pathophysiology of oxidative stress-associated diseases. Thus, reactive oxygen species and reactive nitrogen species (ROS and RNS) were identified as key players in initiating, mediating, and regulating the cellular and biochemical complexity of oxidative stress either as physiological (acting pro-hormetic) or as pathogenic (causing destructive vicious circles) processes. The papers published in this particular Special Issue of *Cells* show an impressive range on the pathophysiological relevance of ROS and RNS, including the relevance of second messengers of free radicals like 4-hydroxynonenal, allowing us to assume that the future will reveal even more detailed mechanisms of their positive and negative effects that might improve the monitoring of major modern diseases, and aid the development of advanced integrative biomedical treatments.

For decades, free radicals were mostly considered as harmful molecules that contribute to the toxic, mutagenic, and carcinogenic bioactivities of different chemical and physical stressors. However, after hydrogen peroxide and nitric oxide were found to have multiple, often cell-type and dose-dependent effects, the new era of interdisciplinary molecular biosciences and translation medicine made significant progress in studies on the pathophysiology of oxidative stress and associated disorders. Therefore, the major goal of this Special Issue of *Cells* is to cover broad aspects of these important scientific areas, still focusing on the cellular level. However, since many disorders are based on altered cellular functions involving interactions of reactive oxygen species (ROS) and reactive nitrogen species (RNS) with macromolecules, in this Special Issue we also try to tackle physiological and pathological aspects of cellular ROS and RNS related to specific cellular processes affecting the entire organism.

While ROS have plenty of physiological activities, particularly in redox signaling and growth regulation, the most aggressive oxygen free radical, which is attributed to the majority of the negative effects of oxidative stress, is hydroxyl radical (HO^.^), known for its ability to damage almost any biomolecule, induce lipid peroxidation, and cause DNA strand breaks [1,2]. Hence, C. Chatgilialoglu et al. presented the chemical, analytical, biological, and diagnostic significance of cyclopurine lesions (cPu) in DNA damage caused by HO^.^, and also alternative damage by UV irradiation [3]. They found the use of cPu lesions as candidate biomarkers of DNA damage, since they are relatively stable and not associated with frequent artifacts like other oxidatively generated DNA lesions. Although rather demanding, LC–MS/MS seems to be the most convenient analytical method for the cPu. C. Chatgilialoglu et al. reveal cPu as a cellular DNA damage biomarker, which is convenient to study in carcinogenesis, aging, and xeroderma pigmentosum.

The very recent review paper of A. Cherkas et al. [4] offered new perspectives to evaluate the importance of glucose not only for the overall cellular oxidative metabolism but also for the overall regulation of the cellular adaptation to stressors, especially to oxidative stress. Going in a similar direction, the K. Gall Trošelj’s group evaluated nutritional stress disturbing the cellular redox-status, increasing the ROS production. In particular, they studied the NRF2-NQO1 axis, which represents a protective mechanism against oxidative stress. Their study involved different cancer cell lines (FaDu, Cal 27, and Detroit 562), which have different basal NQO1 activity and were exposed to the absence of glucose and glutamine, or solely to the low levels of either glucose or glutamine [5]. While all the cells lines analyzed showed sensitivity to glucose deprivation, their differential activation of the NRF2-NQO1 axis resulted in a differently increased expression of NQO1. Therefore, further exploration of such stress response in complex biological systems, focusing in particular on NQO1*2/*2 polymorphism (rs1800566), is required. 

A. Methner’s group studied in vitro mitochondrial structure and function, including ATP content as well as mitochondrial quality control to study mild oxidative stress in a model of Charcot–Marie tooth disease, which is a hereditary polyneuropathy caused by mutations in Mitofusin-2 (MFN2), a GTPase in the outer mitochondrial membrane involved in the regulation of mitochondrial fusion and bioenergetics [6]. By using MFN2-deficient fibroblasts stably expressing wildtype or R94Q MFN2 they found that the mutation R94Q in MFN2 decreases ATP production but increases mitochondrial respiration under conditions of mild oxidative stress. This was associated with an increase of glucose uptake and an upregulation of hexokinase 1 and pyruvate kinase M2, suggesting increased pyruvate shuttling into mitochondria, resulting in mitophagy due to the less efficient mitochondrial quality control in mitochondria of R94Q cells.

Increased sensitivity to oxidative stress was also reported for the human neuroblastoma SH-SY5Y cells upon increased TRPM2 channel expression, as reported by X. An et al. [7]. Namely, SH-SY5Y cells are used as in vitro study models for neurodegenerative diseases, as recent finding showed that the 1-methyl-4-phenylpyridine ion (MPP), which selectively causes dopaminergic neuronal death leading to Parkinson’s disease-like symptoms, can reduce SH-SY5Y cell viability by inducing H2O2 generation and subsequent TRPM2 channel activation. After establishing the stable SH-SY5Y cell line overexpressing the human TRPM2 channel, the authors observed augmented H2O2-induced cell death rates and a reduction in cell viability, which were prevented either by 2-APB, a TRPM2 inhibitor, or by PJ34 and DPQ, poly(ADP-ribose) polymerase (PARP) inhibitors. Therefore, they concluded that TRPM2 channel plays a critical role in conferring the ROS-induced death of SH-SY5Y cells. 

The relevance of oxidative stress and advantages/disadvantages of different experimental models used, especially in vitro, to study human pathophysiology was further stressed by O. Alamu et al., who analyzed the relevance of differential sensitivity of different endothelial cell lines to hydrogen peroxide used for in vitro studies of the blood–brain barrier (BBB) [8]. The authors found that even standardized and well-known cell lines can substantially differ in their antioxidant characteristics. In accordance, they recommend caution in making comparisons across BBB models utilizing distinctly different cell lines that require further prerequisites to ensure that in vitro BBB models involving these cell lines are reliable and reproducible.

In the review on the roles of toll-like receptors (TLRs) in nitroxidative stress, Y. Li et al. focus mostly on the TLR2 and TLR4 in the innate immunity cells to conclude that TLRs trigger a signaling cascade, resulting in the generation of ROS and RNS [9]. Namely, TLRs stimulate immune competent cells to produce pro-inflammatory factors upon pathogen invasion, which can induce oxidative stress. Moreover, TLRs can also affect antioxidant mechanisms that attenuate regulate oxidative stress. Hence, the signaling pathways of TLRs can upregulate the expression of cytokines and RSO and RNS production through inflammatory cells.

Going a step further, M. Jaganjac et al. described the important roles of the lipid peroxidation product acrolein and NADPH oxidase in the granulocyte-mediated growth-inhibition of tumor cells [10]. Namely, in a series of research papers published several years ago, these authors dealt with the phenomenon of spontaneous cancer regression using mostly murine tumors, W256, EAT, and melanoma B16 [11,12]. Eventually, they revealed that the oxidative burst of granulocytes is responsible for spontaneous regression of these neoplasms, while the crucial element for the achieved cancer regression is cytotoxic reactive aldehydes generated by lipid peroxidation and myeloperoxidase, in particular 4-hydroxynonenal (4-HNE) and acrolein. In their study, they analyzed the involvement of reactive aldehydes in cellular redox homeostasis and surface TLR4 expression. They found acrolein to act as an inducer of the granulocyte TLR4 expression, while granulocyte-mediated antitumor effects were shown to be mediated via HOCl intracellular pathway by the action of NADPH oxidase. Thus, they confirmed the interference of intracellular inflammatory signaling pathways of TLRs and oxidative stress, which require further studies to understand the interaction between TLR4 and granulocyte-tumor cell intercellular signaling pathways.

The interference of oxidative stress and cellular inflammatory pathways were also studied by A. Jastrab et al., who produced a study on the expression of inflammatory proteins in keratinocytes [13]. Namely, exposure to UV light is a known causative factor in acute skin photodamage, chronic photoaging, and skin carcinogenesis, although it can also be beneficial for the treatment of chronic inflammatory diseases, such as psoriasis [14]. Due to frequent exposure of almost any person to solar UV irradiation and the ongoing increase in skin cancer incidence (especially of melanoma) there is a lot of interest to define convenient protective/antioxidant substances that could prevent undesirable effects of UV light on skin cells. Hence, the authors analyzed, in vitro, the potentially beneficial effects of cannabidiol (CBD), a natural phytocannabinoid without psychoactive effects, which is a well-known anti-inflammatory and antioxidant substance. They found CBD to significantly enhance antioxidant enzymes such as superoxide dismutase and thioredoxin reductase in UV irradiated keratinocytes, while it reduced the levels of glutathione. CBD also reduced lipid peroxidation, as observed by decreased levels of 4-HNE and 15d-PGJ2. Moreover, CBD influenced interactions of transcription factors Nrf2-NFκB by inhibiting the NFκB pathway, increasing the expression of Nrf2 activators. Thus, the antioxidant activity of CBD through Nrf2 activation as well as its anti-inflammatory properties as an inhibitor of NFκB suggests that CBD could be a useful skin protective agent. 

The association of inflammatory factors, such as histamine, and the onset of oxidative stress affecting the blood vessels was studied by P. Avdonin et al. [15]. In particular, the authors studied effects of NAD(P)H oxidase (NOX) inhibitor VAS2870 (3-benzyl-7-(2-benzoxazolyl)thio-1,2,3-triazolo[4,5-d]pyrimidine) on the histamine-induced elevation of free cytoplasmic calcium concentration ([Ca^2+^]i) and the secretion of vonWillebrand factor (vWF) in human umbilical vein endothelial cells (HUVECs), and on the relaxation of rat aorta in response to histamine. They found that VAS2870 attenuated histamine-induced secretion of vWF, although it did not inhibit its basal secretion. However, VAS2870 did not change the degree of histamine-induced relaxation of rat aortic rings constricted by norepinephrine. In accordance, the authors suggest that NOX inhibitors might be useful as a tool for preventing deep vein thrombosis induced by histamine release from mast cells without affecting vasorelaxation, which, of course, requires intense additional studies, both in vitro and in vivo.

The association between oxidative stress and the functional activities of blood vessels is usually addressed in terms of tackling the onset and progression of atherosclerosis. Therefore, S. Fiorelli et al. analyzed the oxidative stress from the aspects of the Nrf2/HO-1 axis in monocyte-derived macrophages (MDMs) obtained from healthy subjects and from patients with coronary artery disease (CAD), in relation to coronary plaque features evaluated in vivo by optical coherence tomography (OCT) [16]. They found that the MDMs of healthy subjects exhibited a lower oxidative stress status, lower Nrf2, and HO-1 levels as compared to CAD patients. High HO-1 levels in MDMs were associated with the presence of a higher macrophage content, a thinner fibrous cap, and a ruptured plaque with thrombus formation, as detected by OCT analysis. Thus, by revealing the activation of Nrf2/HO-1 pathways as an antioxidant response mechanism in MDMs in CAD patients, the authors suggest that HO-1 levels may reflect coronary plaque vulnerability. They assume that evaluating this association could help in the identification of patients with rupture-prone plaque and suggest Nrf2/HO-1 pathways as a new potential therapeutic targets to counteract plaque progression.

The frequent consequences of atherosclerotic changes affecting coronary blood vessels are ischemia and reperfusion of the heart, i.e., induced oxidative stress of the cardiac muscle cells. However, there are indices suggesting that short-term intermittent hypoxia (IH), similar to ischemia preconditioning, could be beneficiary, and even cardioprotective. This phenomenon was focused on in the research produced by K.T. Young’s group [17]. Aiming to find out if IH exposure can enhance the antioxidant capacity of the heart muscle cells, acting as a cardioprotective mechanism against oxidative stress and ischemia/reperfusion (I/R) injury in vitro, they cultured primary rat neonatal cardiomyocytes under IH condition with an oscillating O2 concentration between 20% and 5% every 30 min. They found that IH protected cardiomyocytes against H2O2- and I/R-induced cell death, most likely because H2O2-induced Ca^2+^ imbalance and mitochondrial membrane depolarization was attenuated by IH, which also reduced the I/R-induced Ca^2+^ overload. Moreover, IH increased the expression of superoxide dismutase (SOD), notably of Cu/Zn SOD and Mn SOD, the total antioxidant capacity, and the activity of catalase, suggesting that IH may indeed protect the cardiomyocytes against H2O2- and I/R-induced oxidative stress, maintaining Ca^2+^ homeostasis as well as the mitochondrial membrane potential and upregulation of antioxidant enzymes.

The last paper of this Special Issue of *Cells* deals with novel and challenging topic of oxidative stress modulation as an option to regulate the healing of soft-tissue wounds or even bone fractures [18]. Thus, E. Skrzydlewska’s group described the beneficial effects of vitamins K and D3 on the redox homeostasis of human osteoblasts if cultured in the presence of hydroxyapatite-based biomaterials [19]. The authors observed that hydroxyapatite-based biomaterials induce oxidative stress manifested by the increased production of reactive oxygen species and decreased glutathione levels and glutathione peroxidase activity, causing lipid peroxidation manifested by an increase of 4-HNE levels, which is known to influence the growth of bone cells [20]. Thus, they confirmed previous findings on the importance of 4-HNE as a regulatory factor that affects bioactivities of the biomaterials in vitro, and might eventually explain at least some of the activity principles of enhanced fracture healing upon insertion of bioactive glass [21]. In the study presented by E. Ambrozewicz et al., vitamins D3 and K were shown to help maintain redox balance and prevent lipid peroxidation in osteoblasts cultured with hydroxyapatite-based biomaterials promoting the growth of the osteoblasts [19]. 

## Conclusions

Observed together, the papers published in this particular Special Issue of *Cells* show an impressive range on the pathophysiological relevance of ROS and RNS, including the relevance of second messengers of free radicals like 4-HNE, allowing us to assume that the future will reveal even more detailed mechanisms of their positive and negative effects that might improve the monitoring of major modern diseases and the development of advanced integrative biomedicine treatments. 

## References

[1] Zarkovic N. (2018). Antioxidants and Second Messengers of Free Radicals. Antioxidants.

[2] Zarkovic K., Jakovcevic A., Zarkovic N. (2018). Contribution of the HNE-Immunohistochemistry to Modern Pathological Concepts of Major Human Diseases. Free Radic. Biol. Med..

[3] Chatgilialoglu C., Ferreri C., Geacintov N.E., Krokidis M.G., Liu Y., Masi A., Shafirovich V., Terzidis M.A., Tsegay P.S. (2019). 5′,8-Cyclopurine Lesions in DNA Damage: Chemical, Analytical, Biological, and Diagnostic Significance. Cells.

[4] Cherkas A., Holota S., Mdzinarashvili T., Gabbianelli R., Zarkovic N. (2020). Glucose as a major antioxidant: When, what for and why it fails?. Antioxidants.

[5] Milković L., Tomljanović M., Čipak Gašparović A., Novak Kujundžić R., Šimunić D., Konjevoda P., Mojzeš A., Đaković N., Žarković N., Gall Trošelj K. (2019). Nutritional Stress in Head and Neck Cancer Originating Cell Lines: The Sensitivity of the NRF2-NQO1 Axis. Cells.

[6] Wolf C., Zimmermann R., Thaher O., Bueno D., Wüllner V., Schäfer M.K.E., Albrecht P., Methner A. (2019). The Charcot–Marie Tooth Disease Mutation R94Q in MFN2 Decreases ATP Production but Increases Mitochondrial Respiration under Conditions of Mild Oxidative Stress. Cells.

[7] An X., Fu Z., Mai C., Wang W., Wei L., Li D., Li C., Jiang L.H. (2019). Increasing the TRPM2 Channel Expression in Human Neuroblastoma SH-SY5Y Cells Augments the Susceptibility to ROS-Induced Cell Death. Cells.

[8] Alamu O., Rado M., Ekpo O., Fisher D. (2020). Differential Sensitivity of Two Endothelial Cell Lines to Hydrogen Peroxide Toxicity: Relevance for In Vitro Studies of the Blood–Brain Barrier. Cells.

[9] Li Y., Deng S.L., Lian Z.X., Yu K. (2019). Roles of Toll-Like Receptors in Nitroxidative Stress in Mammals. Cells.

[10] Jaganjac M., Matijevic Glavan T., Zarkovic N. (2019). The Role of Acrolein and NADPH Oxidase in the Granulocyte-Mediated Growth-Inhibition of Tumor Cells. Cells.

[11] Žarković N., Živković M., Schaur R.J., Poljak Blaži M., Žarković K. Method of Obtaining Granulocytes as Bioactive Agent Able to Inhibit the Growth of Tumor Cells. WO2009077794 (A2), 2007 (Patent Pending). https://register.epo.org/application?number=EP08862465&tab=main.

[12] Jaganjac M., Cipak A., Schaur R.J., Zarkovic N. (2016). Pathophysiology of Neutrophil-mediated Extracellular Redox Reactions. Front. Biosci. (Landmark Ed.).

[13] Jastrząb A., Gęgotek A., Skrzydlewska E. (2019). Cannabidiol Regulates the Expression of Keratinocyte Proteins Involved in the Inflammation Process through Transcriptional Regulation. Cells.

[14] Ambrożewicz E., Wójcik P., Wroński A., Łuczaj W., Jastrząb A., Zarkovic N., Skrzydlweska E. (2018). Pathophysiological Alterations of Redox Signaling and Endocannabinoid System in Granulocytes and Plasma of Psoriatic Patients. Cells.

[15] Avdonin P.V., Rybakova E.Y., Avdonin P.P., Trufanov S.K., Mironova G.Y. (2019). VAS2870 Inhibits Histamine-Induced Calcium Signaling and vWF Secretion in Human Umbilical Vein Endothelial Cells. Cells.

[16] Fiorelli S., Porro B., Cosentino N., Di Minno A., Manega C.M., Fabbiocchi F., Niccoli G., Fracassi F., Barbieri S., Marenzi G. (2019). Activation of Nrf2/HO-1 Pathway and Human Atherosclerotic Plaque Vulnerability: An In Vitro and In Vivo Study. Cells.

[17] Chang J.C., Lien C.F., Lee W.S., Chang H.R., Hsu Y.C., Luo Y.P., Jeng J.-R., Hsieh J.-C., Yang K.-T. (2019). Intermittent Hypoxia Prevents Myocardial Mitochondrial Ca^2+^ Overload and Cell Death during Ischemia/Reperfusion: The Role of Reactive Oxygen Species. Cells.

[18] Mouthuy P.A., Snelling S.J.B., Dakin S.G., Milković L., Čipak Gašparović A., Carr A.J., Žarković N. (2016). Biocompatibility of implantable materials: An oxidative stress viewpoint. Biomaterials.

[19] Ambrożewicz E., Muszyńska M., Tokajuk G., Grynkiewicz G., Žarković N., Skrzydlewska E. (2019). Beneficial Effects of Vitamins K and D3 on Redox Balance of Human Osteoblasts Cultured with Hydroxyapatite-Based Biomaterials. Cells.

[20] Milkovic L., Cipak Gasparovic A., Zarkovic N. (2015). Overview on major lipid peroxidation bioactive factor 4-hydroxynonenal as pluripotent growth regulating factor. Free Radic. Res..

[21] Milkovic L., Hoppe A., Detsch R., Boccaccini A.R., Zarkovic N. (2014). Effects of Cu-doped 45S5 bioactive glass on the lipid peroxidation-associated growth of human osteoblast-like cells in vitro. J. Biomed. Mat. Res. Part A.

